# Sir T. Lauder Brunton on Arsenic and Alcohol

**Published:** 1901-05-11

**Authors:** 


### SIR T. LAUDER BRUNTON ON ARSENIC AND ALCOHOL.

In an important statement on the action of arsenic made to the Royal Commission on arsenical poisoning, Sir T. Lauder Brunton threw out various suggestions as to the possible modifications in action and effect which may result from the combination, or the simultaneous administration of alcohol and arsenic, which are of considerable interest. He says that during the circulation of arsenic in the blood it is not improbable that its action may be determined to one part of the body or another by other substances taken at the same time. A well-marked instance of this kind of action is known, he says, in the case of mercury. Alcohol has a strong affinity for the nervous structures in the body, whilst that of mercury is much less. But alcohol seems to direct mercury to the nervous structures, and causes it to act upon them. An example of this is given by the fact that during the preparation of an alcoholic compound of mercury (mercuric methide) two chemists who were subjected to the fumes were poisoned by the compound and showed signs of nervous disease, one of them becoming maniacal and dying within three months, while the other became idiotic and died after about a year's confinement in a lunatic asylum. Osler, again, has observed that mercury taken along with alcohol seems to have more tendency to produce neuritis than if taken alone, so that it is by no means impossible that some analogous influence may have been in operation in the arsenical beer poisoning in Manchester. At any rate, it is worthy of note, as stated by Sir Lauder Brunton, that in an epidemic of arsenical poisoning at Würzburg, where the arsenic was contained in bread, only one case of paralysis was observed in 373 cases of poisoning. This extraordinary want of action on the motor nerves when taken in bread, as compared with its action when taken along with alcohol in beer or wine, tends strongly to confirm the hypothesis he puts forward.

It may be, however, as we have suggested in these columns, that the arsenic may have been present in the beer in the form of some organic combination of specially injurious activity. As to this Sir Lauder Brunton points out that if the statements made by some of the patients in the recent epidemic as to the quantity of beer they had taken are correct, the amount of arsenic consumed in some cases must have been so exceedingly small as to lead one to ask whether the neuritis can have been due to arsenic alone, even though its action may have been directed to the nerves by alcohol or hops, or whether some other poison besides arsenic has not been present; or, lastly, whether the arsenic may not have been present in the beer in such a combination as to have had a much more deleterious action than pure arsenic. It is quite possible, he says, that arsenic may be present in beer combined with either a hydrocarbon or a proteid, for it has been found to enter into combination with casein. The whole research in regard to this question is full of difficulties, but Sir Lauder Brunton and Professor Hewitt are now engaged upon it, and the results will be awaited with much interest.

